# Acknowledgement to Reviewers of *Proteomes* in 2017

**DOI:** 10.3390/proteomes6010004

**Published:** 2018-01-23

**Authors:** Proteomes Editorial Office

**Affiliations:** MDPI AG, St. Alban-Anlage 66, 4052 Basel, Switzerland

Peer review is an essential part in the publication process, ensuring that *Proteomes* maintains high quality standards for its published papers. In 2017, a total of 33 papers were published in the journal. Thanks to the cooperation of our reviewers, the median time to first decision was 25 days and the median time to publication was 67 days. The editors would like to express their sincere gratitude to the following reviewers for their time and dedication in 2017:
Achour, BrahimMagnus, BackAn, JieMalmström, LarsAn, KyungehMartins, Ian JamesAndrieux, AnnieMaserti, Bianca ElenaAntonio Speretto, RaulMasselon, Christophe D.Aryal, Uma K.Mattei, BenedettaBaran, JaroslawMcDonagh, BrianBenndorf, DirkMedina Piles, VicenteBrakhage, AxelMeisrimler, Claudia-NicoleBrinchmann, Monica F.Mesuere, BartByrne, BernadetteMolassiotis, AthanassiosCalvete, Juan J.Moro, LoredanaCarmine, GuarinoMuddiman, DavidChang, Ing-FengNaryzhny, StanislavChen, Yu-JuObrępalska-Stęplowska, AleksandraChen, Chao-JungOjima, KoichiCologna, StephanieOKUMURA, NobuakiCramer, RainerPadula, MatthewCsősz, ÉvaParnell, Andrew C.Dehzangi, Abdollah (Iman)Parsons, HarrietDjukanovic, RatkoPawłowski, Tomasz A.El-Maarouf-Bouteau, HayatPerez, LaurentEspen, LucaPuglia, Anna MariaFinnie, ChristineRakus, DariuszFischer, RomanRandall, Stephen K.Frost, DustinReinders, JoergGelfi, CeciliaSakai, AtsushiGozes, IllanaSalomon, ArthurGreene, Lesley H.Sancho, JaimeHalvorsen, Trine GrønhaugSathe, Gajanan JalindarnathHelm, RichardSchrama, DeniseHsuan, J.Shui, Hao-aiHuang, Wen-LiiShukla, Hem D.Iraci, NunzioSinha, RaghuJagtap, PratikSpencer, Charles T.Jahn, OlafStodghill, PaulJehmlich, NicoSudhir, Putty-ReddyJob, DominiqueTakahisa, KanekiyoKatsoris, PanagiotisTarro, GiulioKobeissy, Firas H.Tyan, Yu-ChangKoh, Chung-YanVergara, DanieleKosova, KlaraVermeulen, MichielKrüger, MarcusVitorino, RuiLaakkonen, Eija K.Wang, Wei-QingLalowski, MaciejWöhlbrand, LarsLan, PingWoo, Sun HeeLin, Kwang-HueiZhang, WeiLiu, Chun-JieZhang, YixiangLockridge, OksanaZhe, ChengLudwig-Müller, JuttaZivy, MichelLuthra, Amit


